# Exploratory and confirmatory factor analysis of emPHasis‐10: The health‐related quality‐of‐life measure in pulmonary hypertension

**DOI:** 10.1002/pul2.12378

**Published:** 2024-05-12

**Authors:** Gregg H. Rawlings, Chris Gaskell, Nigel Beail, Andrew Thompson, Iain Armstrong

**Affiliations:** ^1^ Clinical and Applied Psychology Unit University of Sheffield Sheffield UK; ^2^ Department of Neuropsychology North Staffordshire Combined NHS Foundation Trust Stoke‐on‐Trent UK; ^3^ South Wales Clinical Psychology Training Programmes, Cardiff and Vale University Health Board Cardiff University Cardiff UK; ^4^ Sheffield Pulmonary Vascular Disease Unit, Sheffield Teaching Hospitals NHS Foundation Trust Royal Hallamshire Hospital Sheffield UK

**Keywords:** functional ability/impairment/quality of life/physical activity, prevention, pulmonary arterial hypertension, pulmonary hypertension

## Abstract

The emPHasis‐10 is a health‐related quality of life (HRQoL) unidimensional measure developed specifically for adults with pulmonary hypertension. The tool has excellent psychometric properties and is well used in research and clinical settings. Its factor structure has not been examined, which may help to identity a complimentary approach to using the measure to examine patient functioning. We performed an exploratory factor analysis (EFA) and confirmatory factor analysis (CFA) on a data set collected from 263 adults with PH recruited from a community setting. The EFA suggested the emPHasis‐10 consists of three underlying latent variables, which based on the loading of items, were termed “fatigue” (Items 3, 4, and 5), “independence” (Items 7, 8, 9, and 10), and “breathlessness” (Items 1, 2, and 6). All factors were found to have good internal consistency. “Independence” accounted for most of the variance (29%), followed by “breathlessness” (22%) and “fatigue” (19%). The CFA looked to confirm the fit of a three‐factor model. A higher‐order model was found to be the best fit consisting of HRQoL as a superordinate factor, for which the association between this factor and the 10 items was mediated through the three latent factors. Further analyses were performed testing the validity of the latent variables revealing all were significantly correlated with self‐reported measures of depression, anxiety, health‐anxiety, and dyspnea. Our analyses support the emPHasis‐10 as a measure of HRQoL, while also proposing the clinical utility of examining the three emergent factors, which could be used to glean additional insight into the respondent's functioning and inform care.

## INTRODUCTION

Pulmonary hypertension (PH) is a group of serious and life‐limiting diseases, which are associated with high rates of early mortality^1^, ^2^ and morbidity.^3^, ^4^ PH is characterized by elevated pulmonary artery pressure at rest.^5^ Symptoms of PH can include dyspnea, fatigue, weakness, reduced exercise tolerance, near‐syncope, pain and edema.^6^ The condition is also associated with high rates of psychological difficulties, including anxiety and depression.^7^ Most forms of PH cannot be cured and as such, treatment aims to prolong life, reduce functional impairment and symptom burden,^8^ and promote health‐related quality of life (HRQoL).

Indeed, there is a body of evidence demonstrating the detrimental impact of PH on HRQoL in adults.^9^, ^10^, ^11^, ^12^ Many of these studies have used the emPHasis‐10 to measure HRQoL. This is a short, disease specific patient reported outcome measure that can easily be administered in clinical and research settings, and which is simple to score and interpret.^10^ Scores on the emPHasis‐10 correlate with a range of self‐reported outcomes^13^, ^14^, ^15^, ^16^, ^17^, ^18^ and objective measures. For instance, the measure has been shown to be an independent predictor of mortality^19^ and can discriminate between patients with PH stratified by World Health Organization (WHO) functional class (Class 1–4 with a higher class indicative of greater severity of symptoms).^10^ Research has shown that the tool is associated with excellent measurement properties, including test–retest (intraclass correlation coefficient = 0.95) and internal consistency reliability (Cronbach's *α* = 0.9).^10^, ^12^

The emPHasis‐10 was developed using Rasch analysis, which is a method that assumes a set of items may be used to measure a single construct. For example, 32 items were initially assessed, which were reduced to 10 items, which subsequently form the unidimensional measure, emPHasis‐10. In comparison, other commonly used HRQoL measures for people with PH are multidimensional,^12^ meaning they assess HRQoL via multiple factors. The Cambridge Pulmonary Hypertension Outcome Review has four subscales (energy level, edema, breathlessness, and mood)^11^; the Living with Pulmonary Hypertension Questionnaire consists of two (physical and emotional)^20^; and the Psychometric Validation of the Pulmonary Arterial Hypertension‐Symptoms and Impact has two symptom domains (cardiopulmonary symptoms and cardiovascular symptoms) and two impact domains (physical impacts and cognitive/emotional impacts).^21^

The identification of factors underpinning a measure can be an important step in the development and validation of multidimensional measures of health‐, behavioral‐, and social‐related constructs. To achieve this, factor analyses can be performed typically later in the development of a measure once a set of items have been conceived, administered, and evaluated.^22^ A factor analysis is a regression model that examines the possibility of underlying latent variables of a measure and the extent to which the relationships between the items are internally consistent. An exploratory factor analysis (EFA) is typically performed first on a measure if no previous or generally accepted factor structure has been proposed. The initial model identified by the EFA is preliminary and should then be subjected to verification through additional analyses including a confirmatory factor analysis (CFA). In other words, a CFA is a hypothesis‐driven analysis that helps to determine how well data fits the proposed model generated from an EFA.^23^ It is typically after this stage that tests of reliability and validity are performed.^22^

Although the initial Rasch analyses validated the unidimensional model of the emPHasis‐10, it is possible that the measure also consists of multiple factors which are correlated with different items. In fact, there are a range of health‐related measures that generate an overall score, as well provide the option of assessing individual domains via a selection of items.^24^, ^25^, ^26^, ^27^ The clear advantage of this approach is that more can be gleaned about the respondent via further analysis of the different domains without the need to ask additional questions. It is possible to utilize Rasch modeling and exploratory and confirmatory factor analyses in conjunction to explore and validate the same measure, especially when the aim is to reduce a set of items to a smaller number of summary scale scores. However, the order and purpose of this needs to be considered given the analyses underlying assumptions and requirements.^28^, ^29^ Our aim is not to challenge the validation of the emPHasis‐10 as a unidimensional measure but instead, to examine its factor structure recognizing that it may also be examining different domains, which when combined, measure HRQoL and thus supporting the unidimensional model. Such domains have clear relevance in clinical settings as it may help generate a more nuanced understanding of the patient and inform treatment.

The aim of this study was to conduct the first EFA on data collected using the emPHasis‐10 measure. A principal components analysis (PCA) could have been used instead of an EFA as both methods aim to summarize a series of variables into a smaller number of factors or components. Whereas PCA identifies components based on the variance of items, EFA uses the covariance to identify factors. EFA was chosen here as we were more interested in identifying factors based on the covariance of items, recognizing the emPHasis‐10 was initially developed as a unidimensional measure, and factors underlying or explaining the data, rather than looking to reduce the number of items, which is an aim of PCA.^30^ Next, we performed a CFA on the proposed structured of the scale that was identified from the EFA using a split data set. Finally, we explored the validity of the emergent factors by analyzing their relationship with demographic, PH‐specific and self‐reported factors (e.g., anxiety, depression dyspnea) to explore validity.

## METHODS

### Participants

Data were collected across three primary research studies, the key findings from which have already been published.^13^, ^14^, ^17^ Individual studies had received favorable ethics opinion by either the Schools of Business, Law and Social Sciences Research Ethics Committee at Nottingham Trent University (2021/417) or The University of Sheffield (035318 and 034442). All participants provided consent for their data to be used for future research. To be eligible for the current study, participants must have been aged 18 years or older, confirmed that they had been diagnosed with PH (all forms were accepted) by a medical professional, literate in English, able to complete self‐reported questionnaires without help from others, and able to provide informed consent. In one of the samples, participants (*n* = 77) must have been experiencing difficulties with anxiety and not experiencing any thoughts of self‐harm or suicide. In the other two samples (*n* = 186), participants must have been living in the United Kingdom.

An adequate sample is needed to be able to state, with a degree of confidence, that the factor solution can be generalized to the wider population. A range of recommendations have been proposed regarding the necessary sample size for a factor analysis: this has included an absolute threshold ranging from 100 to over 1000 participants, a ratio of 3:20 times the number of variables investigated or a sample size based on the variables‐to‐factors ratio and degree of communality.^31^ A systematic review of published factor analyses involving 1750 articles found 89% of sample sizes were over 100—it also highlighted that sample sizes were inflated by studies including students, where sample sizes tended to be greater.^32^ In total, the data from 263 adults with PH recruited from a community setting was examined. Participants were recruited from international PH organizations, specifically PHA UK.

### Measures

All data were collected via questionnaires hosted by Qualtrics for the primary research studies.

#### Demographic and clinical information

Participants were asked to self‐report their demographic (age, sex, ethnicity, country of residence, and employment status) and clinical status (PH diagnosis, WHO functional class, years since being diagnosed with PH).

#### emPHasis‐10

This HRQoL measure asks participants to endorse a list of 10 items using a scale of 0‐5. For more information, please see: https://www.phauk.org/pha-uk-resources/emphasis-10-questionnaire/. Scores range from 0 to 50 with a higher score suggestive of lower HRQoL. Participants are asked to rate items in the context of their recent experience of living with PH. All 263 individuals completed a full emPHasis‐10 measure.

#### Depression

As an artifact of previous research designs, all participants had completed the Patient Health Questionnaire‐9 (PHQ‐9), which is a measure of depression. This measure consists of nine items relating to depression, which participants are asked to rate on a 4‐item Likert scale. Respondents are asked to consider the symptoms over the last 2 weeks. Higher scores suggest greater symptoms of depression. Cronbach's *α* was good: overall = 0.88, EFA = 0.87, CFA = 0.89.

#### Anxiety

Overall, 142 individuals had completed the Generalized Anxiety Disorder‐7, which is a measure of anxiety. It consists of seven items and as per the PHQ‐9, participants are asked to consider symptoms over the last 2 weeks responding using a 4‐item Likert scale. A greater score is indicative of severe anxiety. Cronbach's *α* was excellent: overall = 0.93, EFA = 0.92, CFA = 0.94.

#### Health anxiety

One hundred and twenty‐one individuals completed the Short‐Form Health Anxiety Inventory, which is a measure of health anxiety. This is a 14‐item measure asking participants about their thoughts, feelings, and behaviors related to health anxiety over the past week using a 4‐item Likert scale is used. Higher score suggests greater health anxiety. Cronbach's *α* was excellent: overall = 0.91, EFA = 0.91, CFA = 0.91.

#### Dyspnea

In total, 77 participants had completed the Dyspnea‐12 (D12), which is a measure of breathing difficulties. Scores range from 0 to 36, with a greater score indicative of more difficulties with breathing. Items are asked in relation to “these days.” A 4‐item Likert scale is used. Cronbach's *α* was excellent: overall = 0.9, EFA = 0.96, CFA = 0.95.

### Data analysis

Data were first cleaned in Microsoft Excel. We used complete case analysis as the data set did not include any missing data. SPSS 28^33^ was used to compare the two groups (EFA vs. CFA group) on demographic and clinical factors. Statistical analyses for the factor analyses were conducted using R (version 4.2.1).^34^ Descriptive statistics and data cleaning was performed using the*Tidyverse* package.^35^ Analysis‐specific packages were used for performing correlations and EFA,^36^ CFA,^37^ path model diagrams,^38^ and data tabulation.^39^ Inspection of the correlation matrix and check of model assumptions were performed. We entered unstandardized data, because the raw data were available.

The data file was initially split using randomization in Microsoft Excel to provide EFA (*n* = 131) and CFA samples (*n* = 132). To achieve this, participants were allocated a random number using a formula in Microsoft Excel. The full list was then ordered in size according to this number. The first half were allocated to the EFA group and second to the CFA group. Inter‐item correlations were performed for the EFA sample using Pearson's method. The Kaiser–Meyer–Olkin and Bartlett's test of sphericity (threshold of 0.7) was used to check the suitability of data for factor analysis. Due to the availability of raw data, this was entered into the model as opposed to correlation/covariance matrices. Due to the suspected relationship among latent variables within emPHasis‐10 an oblique rotation (oblimin) was selected with a maximum likelihood factoring method. In determining the number of factors from the EFA Kaiser's method (above 1.0) and analysis of the elbow joint on the accompanying scree plot was used.

For the CFA, model selection was informed by results from the EFA. In addition, second‐order models (e.g., higher order, bifactor) were considered if indicated by the EFA (e.g., markedly high inter‐factor correlations)—which is also consistent with our understanding of the emPHasis‐10 as a unidimensional measure.

In determining the model of best fit, several goodness‐of‐fit indices were employed. This included the standardized root mean square residual (SRMR), the root mean square error of approximation (RMSEA), the comparative fit index (CFI), and the Tucker–Lewis Index. Thresholds for model suitability included SRMR ≤ 0.08, RMSEA ≤ 0.05, and CFI and NFI ≥ 0.90. When discriminating between multiple models of acceptable fitm preference was given to more parsimonious models (fewer parameters) and smaller values for model Akaike and Bayesian Information Criterion.

Final models were visualized using path diagrams. Latent factors identified by the EFA and CFA were assessed for internal consistency (standardized Cronbach's *α*) and inter‐factor correlation.

## RESULTS

### Participants

A summary of participant's demographics, PH‐related factors, and clinical measures is displayed in Table 1. There were no significant differences in participant characteristics between the two groups, suggesting the random split was successful. In both groups, most individuals were female, living in the United Kingdom, White, and retired. The most common form of PH was idiopathic. The largest group of individuals did not know or failed to report their WHO functional class. On the group level, both were experiencing mild–moderate symptoms of depression and anxiety.

**Table 1:** Summary of participant's characteristics in those randomized to the EFA and CFA.

	EFA sample (*n* = 131)	CFA sample (*n* = 132)	*p*
Demographics			
Age	*M* = 55.3 (SD = 14.1)	*M* = 54.55 (SD = 15.8)	0.69
Sex			0.11
Female	112 (85.5%)	102 (77.3%)	
Male	18 (13.7%)	30 (22.7%)	
Other	1 (0.8%)	0 (0%)	
Country			0.87
UK	116 (88.5%)	116 (87.9%)	
International	15 (11.5%)	16 (12.1%)	
Employment status			
Employed	37 (29.4%)	41 (31.8%)	0.66
Not employed	34 (27%)	27 (20.9%)	
Retired	52 (41.3%)	59 (45.7%)	
Student	3 (2.4%)	2 (1.6%)	
Ethnicity			0.36
White	112 (85.5%)	112 (86.2%)	
Asian	4 (3.1%)	6 (4.6%)	
Black	0 (0%)	2 (1.5%)	
Latina	2 (1.5%)	0 (0%)	
Mixed	1 (0.8%)	2 (1.5%)	
Not reported	12 (9.2%)	8 (6.2%)	
PH‐specific factors			
PH diagnosis			0.14
Idiopathic PH	52 (39.7%)	55 (41.7%)	
CTEPH	26 (19.8%)	29 (22%)	
CTD	13 (9.9%)	6 (4.5%)	
Congenital PH	7 (5.3%)	15 (11.4%)	
Familial PH	0 (0%)	2 (1.5%)	
Other	12 (9.2%)	12 (9.1%)	
Not reported or not sure	21 (16%)	13 (9.8%)	
WHO functional class			0.32
1	12 (9.2%)	8 (6.1%)	
2	24 (18.3%)	29 (21.2%)	
3	39 (29.8%)	37 (28%)	
4	1 (0.8%)	6 (4.5%)	
Not reported or not sure	55 (42%)	53 (40.2%)	
Years diagnosed with PH	*M* = 7.5 (SD = 7.7) *n* = 131	*M* = 9.35 (SD = 9.85) *n* = 131	0.1
Clinical measures			
Depression	*M* = 9.9 (SD = 6.2)	*M* = 9.9 (SD = 6.6)	0.99
	*n* = 131	*n* = 132	
Anxiety	*M* = 8.6 (SD = 5.6)	*M* = 9.1 (SD = 6.6)	0.64
	*n* = 70	*n* = 72	
D12	*M* = 16 (SD = 10.1)	*M* = 15 (SD = 8.7)	0.65
	*n* = 42	*n* = 35	
Health anxiety	*M* = 15.6 (SD = 7.2)	*M* = 15.2 (SD = 7.5)	0.76
	*n* = 61	*n* = 60	
emPHasis‐10	*M* = 26.5 (SD = 12.7)	*M* = 25.6 (SD = 12.6)	0.54
	*n* = 131	*n* = 132	

*Note*: Values reflect number of participants (*n*) unless stated.

Abbreviations: CFA, confirmatory factor analysis; CTD, connective tissue disease; CTEPH, chronic thromboembolic PH; D12, Dyspnea‐12; EFA, exploratory factor analysis; M, mean; PH, pulmonary hypertension; WHO, World Health Organization.

### EFA

The EFA was conducted on a sample of 131 people with PH. The inter‐item correlations of variables included in the measure are displayed in the correlation matrix (Table 2).

**Table 2:** Correlation matrix of 10 items from the emPHasis‐10.

	Q1	Q2	Q3	Q4	Q5	Q6	Q7	Q8	Q9	Q10
Q1	1.00									
Q2	0.70	1.00								
Q3	0.55	0.56	1.00							
Q4	0.62	0.56	0.77	1.00						
Q5	0.58	0.55	0.55	0.73	1.00					
Q6	0.65	0.60	0.55	0.59	0.56	1.00				
Q7	0.52	0.49	0.47	0.52	0.52	0.34	1.00			
Q8	0.58	0.56	0.62	0.61	0.58	0.47	0.69	1.00		
Q9	0.45	0.49	0.45	0.50	0.56	0.44	0.64	0.67	1.00	
Q10	0.45	0.51	0.54	0.57	0.55	0.39	0.61	0.72	0.69	1.00

Abbreviation: Q, Question from emPHasis‐10.

Bartlett's Test of Sphericity was significant (*χ* ^2^ = 881.40, df = 45, *p* = <0.001) confirming inter‐correlations between variables within the correlation matrix. The Kaiser–Meyer–Olkin factor adequacy produced an overall MSA (measures of sampling adequacy) of 0.91 (range = 0.86–0.93), indicating that a proportion of variance in items may be caused by underlying factors. The communalities ranged from 0.58 to 1.

**Figure 1:**
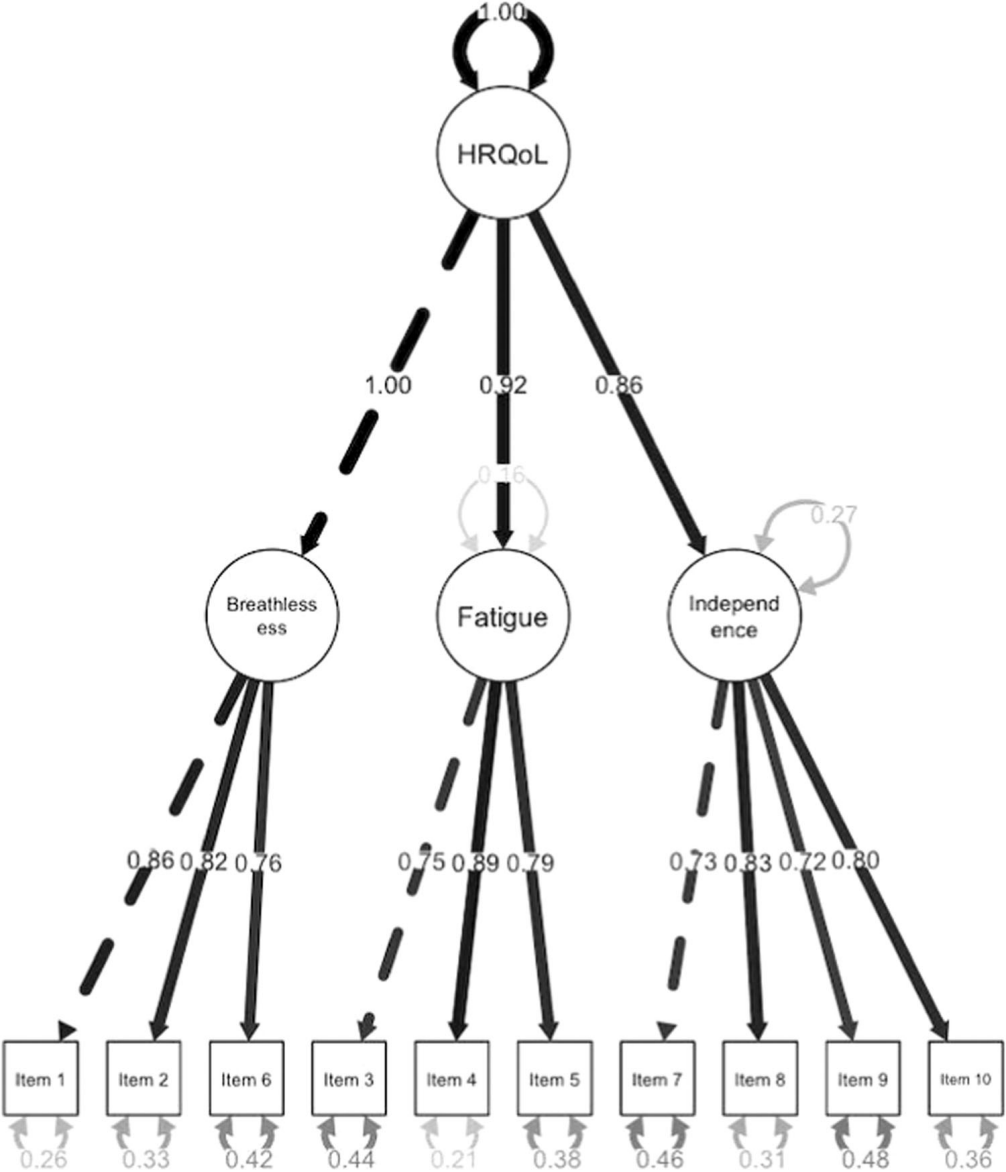
Path model for the higher‐order confirmatory factor analysis. HRQoL, health‐related quality of life.

Analysis of eigenvalues revealed three factors with values above 1.0. This was inconsistent with the elbow point identified from the accompanying scree plot which identified two factors (Supporting Information S1: Figure 1). A three‐factor model was chosen for the initial exploratory factor analysis. This approach made intuitive and clinical sense from the perspective of the authors given the nature of the items. For example, it is conceivable that several items ask about breathing difficulties, some on tiredness and fatigue, and others on the additional burden associated with PH.

The factor loadings are displayed in Table 3. None of the items double‐loaded onto a single factor (<0.4). All but two items were strongly loaded (>0.6). Factor one contained three items (Items 3, 4, and 5). Given the nature of these questions, this factor was named “fatigue.” The standardized Cronbach's *α* found the fatigue factor to have good internal consistency (*α* = 0.87). The consequence of dropping a single item ranged between 0.71 and 0.87. This factor explained the least amount of variance. Factor two contained four items (Items 7, 8, 9, and 10), which appeared to measure “independence.” The Cronbach's *α* found the independence factor to have good internal consistency (*α* = 0.85). Reliability when dropping an item ranged between 0.75 and 0.82. This factor explained the greatest amount of variance. Factor three contained three items (Items 1, 2, and 6). All questions were related to “breathlessness.” The Cronbach's *α* found the breathlessness factor to have good internal consistency (*α* = 0.89). Reliability when dropping an item ranged between 0.85 and 0.87. The variance accounted for by the three factors is shown in Table 3; there was a total of 69% cumulative variance explained by the three‐factor model. The component correlation matrix shows that there were strong correlations among factors based on a sum of items: fatigue and independence (*r* = 0.66), fatigue and breathlessness (*r* = 0.71), and independence and breathlessness (*r* = 0.67).

**Table 3:** Factor loadings for items included in the analysis.

	Factor 1 (Fatigue)	Factor 2 (Independence)	Factor 3 (Breathlessness)	*h* ^2^	*u* ^2^
Q1	–	–	0.86	0.76	1.00
Q2	–	–	0.75	0.66	1.08
Q3	0.57	–	–	0.63	1.27
Q4	1.01	–	–	1.00	1.00
Q5	0.45	–	–	0.61	1.93
Q6	–	–	0.70	0.58	1.13
Q7	–	0.73	–	0.60	1.02
Q8	–	0.75	–	0.75	1.06
Q9	–	0.83	–	0.66	1.01
Q10	–	0.85	–	0.71	1.07
Sum of squared loadings	1.9	2.86	2.18		
Proportion Variance	19.0%	29.0%	22.0%		
Cumulative Variance	69.0%	29.0%	50.0%		
Proportion Explained	27.0%	41.0%	31.0%		
Cumulative Proportion	100.0%	41.0%	73.0%		

Abbreviations: *h* ^2^, commonalities; *u* ^2^, uniqueness.

### CFA

The CFA was conducted on a sample of 132 people with PH. In addition to confirming the fit of the three‐factor model, we sought to compare it to a two‐factor model (as hinted by the scree plot), one‐factor model (given the ten‐items were proposed to measure HRQoL and the high inter‐correlations between factors identified in the EFA), and second‐order models (higher order [suggesting a hierarchical structure whereby first level factors are mediated through a superordinate factor/s] and bifactor [indicating that a general, although separable, factor accounts for a portion of shared variance among all test items]).

In terms of comparing the models, the goodness of fit statistics are shown in Table 4. Overall, the fit for the three‐factor model was sufficient based on the criteria inspected. The likelihood test demonstrated that the three‐factor model produced a significantly greater fit than the two‐factor model (*χ* ^2^ difference = 11.27, df = 34 *p* = 0.004).

**Table 4:** Goodness‐of‐fit statistics of factors.

	One‐factor	Two‐factors	Three/higher	Bifactor
NPAR	20	21	23	30
CHISQ	89.18	49.82	38.55	31.77
DF	35	34	32	25
CFI	93.3%	98%	99.2%	99.2%
TLI	91.4%	97.4%	98.9%	98.5%
AIC	4274.9	4237.5	4230.3	4237.5
BIC	4332.5	4298.1	4296.6	4324.0
RMSEA	0.11	0.06	0.04	0.04
SRMR	0.6	0.04	0.04	0.03
*p*		<0.001	<0.001	0.45

Abbreviations: AIC, Akaike's information criterion; BIC, Bayes information criterion; CFI, comparative fix index; CHISQ, model *χ* ^2^ (deviance); DF, degrees of freedom; NPAR, number of parameters; RMSEA, root mean square error of approximation; SRMR, standardized root mean square residual; TLI, Tucker–Lewis index

When comparing the three‐factor model to the second‐order models, the likelihood test demonstrated that there was no significant difference in fit between the three‐factor model and the bi‐factor model (*χ* ^2^ difference = 6.78, df = 32, *p* = 0.5). The bifactor model was subsequently discarded in favor of the more parsimonious three‐factor model. When comparing a three‐factor model to a higher‐order model in which there are no additional constraints, fit statistics are identical. For these final models, fit statistics were indicative of good model fit.

In terms of choosing between the three‐factor and higher‐order model, the higher‐order model was selected based on theory (that all items included were suspected to contribute towards HRQoL). The inter‐factor correlations between the superordinate “HRQoL” factor and the first‐order factors are shown in the supplementary material (Supporting Information S1: Table 1). The HRQoL factor had good internal consistency (*α* = 0.93). The path model for the higher‐order model is shown in Figure 1, whereas the path model for the three‐factor model is in the Supporting Information materials (Supporting Information S1: Figure 2).

### Relationship with other variables

When comparing a sum of items corresponding to each of the three emergent factors, both fatigue and breathlessness were correlated with WHO functional class, suggesting severity of PH symptoms were related to more fatigue and shortness of breath. As expected, all self‐reported measures of functioning (anxiety, depression, and dyspnea) were significantly correlated with each of the three factors, particularly fatigue (Table 5).

**Table 5:** Correlation matrix between three factors and clinical measures.

	Fatigue	Independence	Breathlessness
PH diagnosis	−0.2	0.01	0.03
WHO functional class	0.24**	0.1	0.22*
Years diagnosed with PH	−0.03	0.07	0.04
Depression	0.56***	0.6***	0.52***
Anxiety	0.48***	0.53***	0.38***
Health‐anxiety	0.42***	0.51***	0.38**
Dyspnea	0.6***	0.39*	0.67***

Abbreviations: PH, pulmonary hypertension; WHO, World Health Organization.

* *p* < 0.05

** *p* < 0.01

*** *p* < 0.001.

## DISCUSSION

There is growing recognition in PH of the importance of including the perspectives of patients in their care.^40^ This can be achieved, in part, through the utilization of patient reported outcome measures including those assessing HRQoL, such as the emPHasis‐10. Indeed, in the United Kingdom where most of the current sample were recruited from, routine assessment of HRQoL is mandatory.^19^ We performed the first factor analytic investigation of the emPHasis‐10. An explorative factor analysis identified a three‐factor higher order model, which was associated with good internal consistency. All 10 items loaded on a factor supporting the initial analyses developing the tool,^10^ which suggested that all items are relevant and contribute to the higher ordinate factor of HRQoL. Moreover, none of the items were cross‐loaded in the current analyses indicating that questions are appropriate in terms of what they are proposed to measure in the current study.

Although intercorrelations were high amongst the 10 items, the CFA suggested that a one‐factor model was insufficient when used to fit the data. This was also somewhat consistent with the observed eigenvalues from the EFA, which suggested at least a two‐factor model. Instead, the analyses revealed the 10 items may represent different aspects of HRQoL in PH in the form of “fatigue,” “breathlessness,” and “independence.” However, it is important to clarify that overall a higher‐order model was most acceptable when fitting the data, which also suggests the association between HRQoL and the 10 items may be mediated through the three superordinate factors. This corroborates the initial validation of the emPHasis‐10 as a unidimensional measure, but also suggests that three subdomains could be identified within the measure. This may add to the clinical utility of the emPHasis‐10 as there is strong evidence to support it being used as a unidimensional measure to examine the individual's overall HRQoL and in conjunction, a score could be calculated for each of the three domains identified here. The additional benefit of using the tool in this way, is that it may lead to a more nuanced understanding of the respondent's overall level of functioning, which could help inform their care, without any additional burden being placed on the respondent. That said, while evidence has demonstrated the emPHasis‐10 is sensitive to identify change associated with treatment, further research is required to examine whether treatment is more effective on specific factors. A further valuable line of inquiry would be to also identify whether meaningful cut off scores, in terms of sensitivity and specificity, could be applied to the emPHasis‐10; for example, recognizing individuals that may be likely to be at risk of experiencing mental health comorbidities. Certainly, given the strong associations between the three factors and measures of distress (anxiety, depression, and dyspnea), it is possible the emPHasis‐10 in some format could be used a proxy measure.

Notwithstanding the factor “independence” consisted of more items (four compared to three items for the other factors), it accounted for the greatest amount of variance (29%) in HRQoL, followed by “breathlessness” (22%) and “fatigue” (19%). This is consistent with the growing evidence supporting the relationship between psychosocial factors and HRQoL in this clinical group in addition to disease‐specific factors.^41^ Although experiences associated with independence have emerged from the qualitative literature examining PH,^6^ it has not been examined specifically and warrants further investigation to ascertain its relationship with other variables. It is important to highlight that the factor breathlessness and scores on the D12 were only moderately correlated, suggesting that, although there is an overlap, they may be investigating different aspects of this symptom. Finally, as identified here, fatigue is a common difficulty in PH explaining nearly one‐fifth of the variance. A study involving 126 individuals with PH found 56% reported their general fatigue as “severe” or “very severe.” Further research is needed to explore fatigue in the context of PH and how best to manage this symptom.^42^

There are several limitations of this study. First, we imposed the terms “independence,” “fatigue,” and “breathlessness” on the factors that emerged. This was based on the content of the items; however, we are aware this is subjective and open to interpretation. The study recruited individuals from the community and while this may help to improve the generalizability of the findings, it may introduce additional biases or limit the type of analyses that can be performed due to missing data. For example, WHO functional class was missing for most participants. Future research using prospective or retrospective data sets collected in clinical settings where patient's records are reviewed for missing values could look to validate the findings of our CFA. More specifically, given an association was observed between WHO functional class and two of the factors, research could stratify individuals depending on their WHO functional class to see if the factor structure is consistent across groups. It is also possible that a greater number of participants could be recruited from such settings recognizing our modest sample size. Indeed, a posthoc review of our sample size, based on the variables‐to‐factors ratio (three to four items per factor) and communality (~0.6 or greater indicting a high level) observed, a sample size of 170 would provide a good‐level criterion—figures were not provided by the authors for an acceptable level.^25^

In summary, this study further demonstrates the solid psychometric characteristics of the emPHasis‐10 and proposes a complimentary approach to using the measure in this clinical group. Our results should be viewed as being in conjunction of research supporting the emPHasis‐10 as a unidimensional measure rather than a contradictory finding. Our factor analyses proposed a high‐order factor structure whereby the 10 items of the emPHasis‐10 and HRQoL are mediated through three factors named “independence,” breathlessness,” and “fatigue.”

## AUTHOR CONTRIBUTIONS

Gregg H. Rawlings developed the concept of the study and was involved in data collection, analysis, and write‐up. He approved the final manuscript for submission. Chris Gaskell was involved in the data analysis and write‐up. He approved the final manuscript for submission. Nigel Beail was involved in data collection and write‐up. He approved the final manuscript for submission. Andrew Thompson was involved in data collection and write‐up. He approved the final manuscript for submission. Iain Armstrong was involved in data collection and write‐up. He approved the final manuscript for submission.

## CONFLICT OF INTEREST STATEMENT

The authors declare no conflict of interest.

## ETHICS STATEMENT

Data were collected across three primary research studies, the key findings from which have already been published.^13^, ^14^, ^17^ Individual studies had received favorable ethics opinion by either the Schools of Business, Law and Social Sciences Research Ethics Committee at Nottingham Trent University (2021/417) or The University of Sheffield (035318 and 034442). All participants provided consent for their data to be used for future research.

## Supporting information

Supporting Information
