# Supplementary material for: Exploratory and confirmatory factor analysis of emPHasis‐10: The health‐related quality‐of‐life measure in pulmonary hypertension
Source: Pulm Circ. 2024 May 12;14(2):e12378. doi: 10.1002/pul2.12378 (PMC11088805; doi:10.1002/pul2.12378)

*Supplementary Figure 1*

*Scree plot identified in the EFA (red line signifies elbow point)*


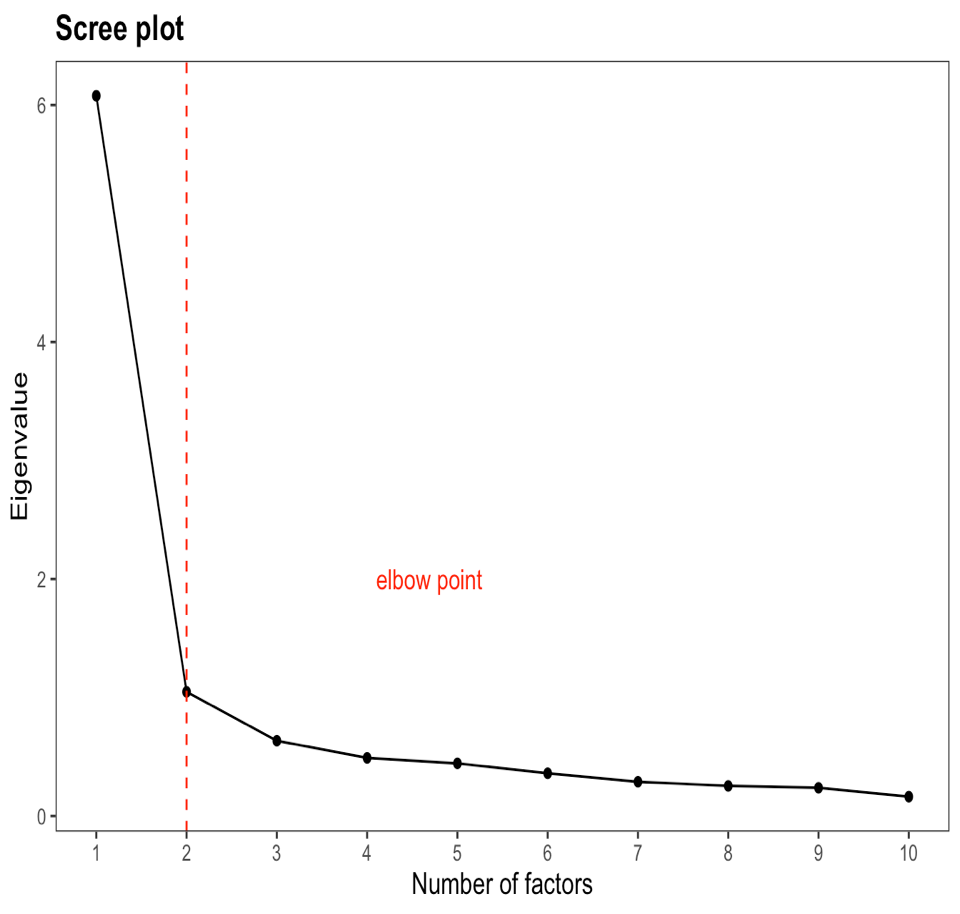


*Supplementary Figure 2*

*Path model for the higher order three-factor confirmatory factor analysis.*


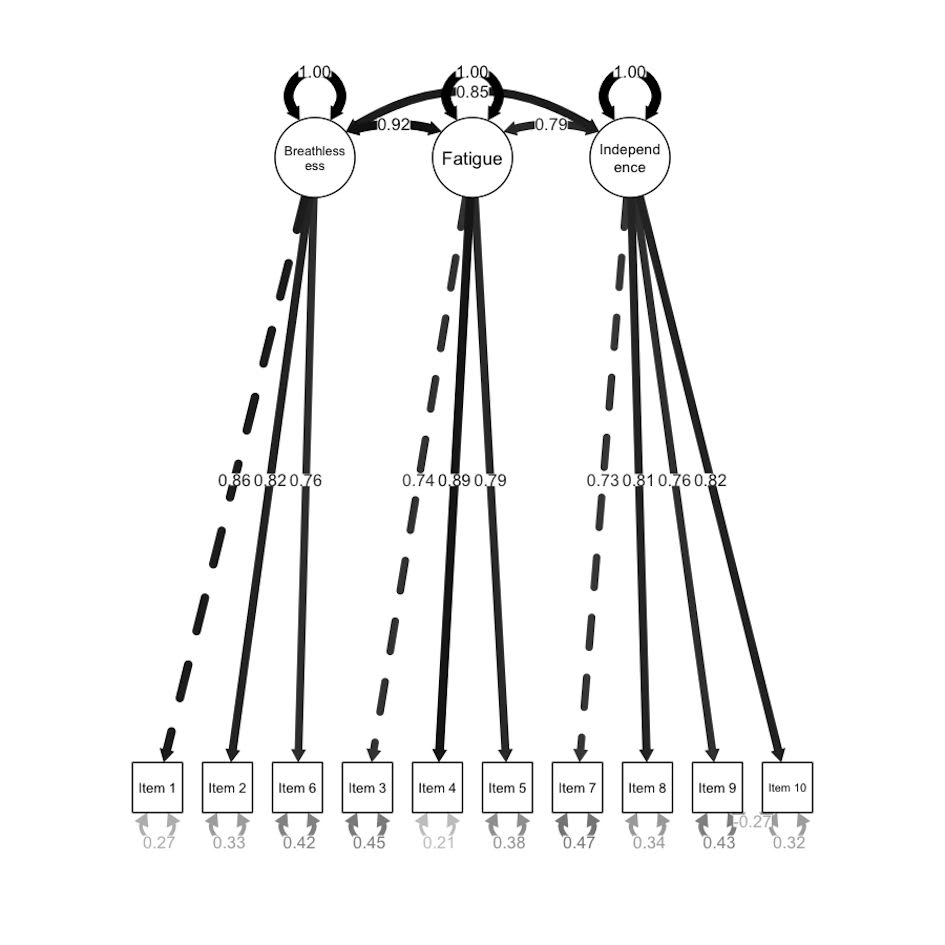


*Supplementary Table 1*

*Standardised loadings for the higher order model of the scale*


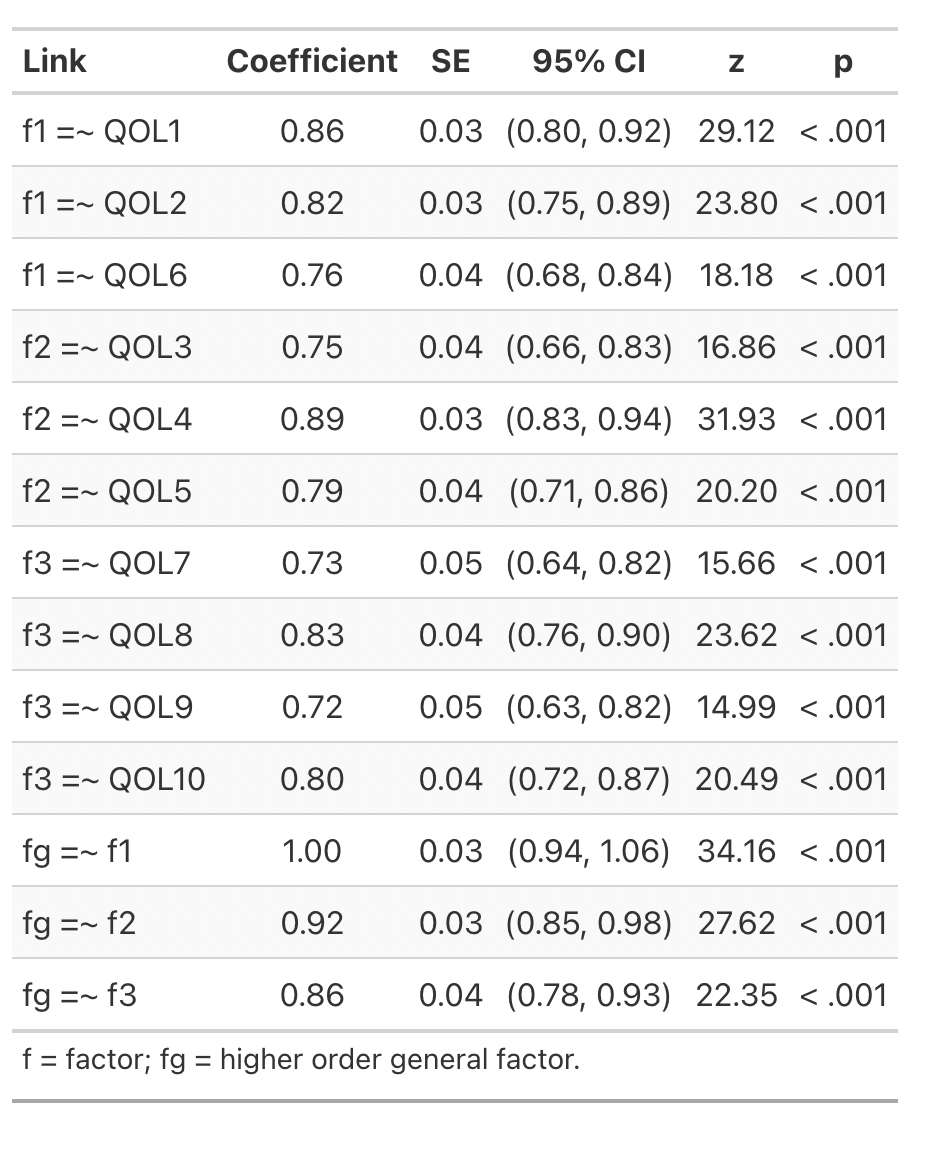

Supplement: Supplementary file 1 — Supporting Information [file PUL2-14-e12378-s001.docx]
